# Hormones, Sexual Function, and Dysfunctional Sexual Beliefs in Postmenopausal Women: A Cross-Sectional Study

**DOI:** 10.3390/jpm16070394

**Published:** 2026-07-22

**Authors:** Clayton Peixoto, Melanie Navarro, Carolina Gomes Carrilho, Antonio José Grande, Antonio Egidio Nardi, Adriana Cardoso, André Barciela Veras

**Affiliations:** 1 Medicine and Psychology Courses, Universidade Estadual de Mato Grosso do Sul, Campo Grande 79115-898, Brazil; 2 Independent Researcher, Campo Grande 79010-780, Brazil; psi.mnavarro@gmail.com; 3 Postgraduate Program in Psychology, Universidade Católica Dom Bosco, Campo Grande 79117-900, Brazil; carolcarrilho1@hotmail.com; 4 Laboratory of Panic and Respiration, Institute of Psychiatry, Federal University of Rio de Janeiro, Rio de Janeiro 21941-902, Brazil; antonioenardi@gmail.com (A.E.N.); barcielaveras@hotmail.com (A.B.V.); 5 Laboratory of Thanatology and Psychiatry in Other Medical Conditions, Institute of Psychiatry (IPUB), Federal University of Rio de Janeiro (UFRJ), Rio de Janeiro 21941-902, Brazil

**Keywords:** sexual desire, sexual dysfunction, sexual beliefs, sex hormones, testosterone, postmenopausal

## Abstract

**Background:** Sexual function in postmenopausal women is influenced by both hormonal and psychological factors. This study investigates the associations between sex hormones, sexual function, and sexual beliefs in this population. **Objective:** This research seeks to assess the relationship between sex hormones, sexual function, and dysfunctional sexual beliefs in postmenopausal women. **Methods:** A cross-sectional study was conducted with 42 postmenopausal women aged 45–65 years. Instruments included the FSFI and SDBQ. Hormones assessed were testosterone, estradiol, progesterone, prolactin, DHEA, SHBG, and LH. Blood samples were collected in the morning and analyzed using chemiluminescence or radioimmunoassay. Pearson correlation tests were used, with Bonferroni adjustment applied to control for multiple comparisons. **Results:** Free testosterone was positively correlated with sexual desire and negatively associated with dysfunctional beliefs regarding sexual desire. Estradiol also showed a positive correlation with desire, while prolactin was negatively associated. No other FSFI domains showed significant hormonal associations. **Conclusions:** Findings suggest that testosterone may influence both sexual desire and the internalization of dysfunctional sexual beliefs in postmenopausal women, highlighting the interplay between biological and psychological dimensions of sexuality, which may be relevant for a more comprehensive clinical assessment of sexual function in this population.

## 1. Introduction

Sexuality in postmenopausal women emerges from a complex interaction of biological, psychological, and sociocultural processes that evolve across the lifespan [1]. While sexual function is often associated with hormonal fluctuations, particularly during the menopausal transition, it is equally shaped by internalized beliefs, cultural norms, and individual psychological factors [2]. Hormonal changes—such as reductions in estrogen and androgen levels—can influence not only physiological aspects of sexual response but also emotional and cognitive dimensions related to intimacy, self-perception, and desire [3,4]. At the same time, personal and socially constructed beliefs about aging, femininity, and the legitimacy of female sexual desire in later life may reinforce or inhibit sexual expression [5]. Understanding this multifaceted relationship is essential for healthcare professionals aiming to promote sexual health and well-being among postmenopausal women.

Postmenopause, defined as the stage following the final menstrual period and marked by ovarian failure, involves profound hormonal changes that affect not only physical health but also mood and quality of life [6,7,8,9,10,11,12]. During this period, estrogen levels fall sharply, contributing to symptoms such as vaginal dryness, genital atrophy, and dyspareunia. Although the ovaries continue to produce androgens to a limited extent, free testosterone levels also tend to decline. Levels below 5–10 ng/dL, for example, have been associated with reduced sexual desire in postmenopausal women [13]. However, due to the complex interplay of physiological and psychosocial variables, it remains difficult to determine the precise role each factor plays; more likely, they influence each other in shaping female sexuality during this stage of life [1].

Beyond physiological factors, the way postmenopausal women experience their sexuality is deeply influenced by internalized beliefs that are often shaped by cultural and religious norms. Dysfunctional sexual beliefs—such as the idea that sexual desire in women is sinful, inappropriate with aging, or exclusively tied to reproductive function—can inhibit sexual expression, even in the presence of physiological capacity [14,15]. These beliefs tend to be reinforced across the life cycle and may become particularly rigid during the postmenopausal period, when social expectations often silence or pathologize female sexuality [15]. As such, these cognitive constructs may act not only as consequences of decreased sexual function but also as mediators or amplifiers of dysfunction [16]. Investigating their relationship with hormonal patterns may help clarify how biological and psychological factors interact to shape sexual experience during this life stage [1].

Given the global increase in female life expectancy, a growing number of women are experiencing postmenopause and becoming more susceptible to health concerns typical of this stage, including sexual dysfunctions. In this context, the present study aims to assess the relationship between sex hormones, sexual function, and dysfunctional sexual beliefs in postmenopausal women, considering the interaction between biological and psychological factors involved in sexual experience.

## 2. Materials and Methods

### 2.1. Participants

We conducted a cross-sectional study for eighteen months, using a convenience sample of 42 women being monitored in an outpatient clinic for climacteric-related complaints in Campo Grande, MS, Brazil. Participants were women aged between 45 and 65 years at the time of the assessment, with a gynecological diagnosis of postmenopause (with 12 or more months since the last menstruation). Women with diabetes, hormone replacement therapy (HRT) or other metabolic conditions that could significantly alter a participant’s hormonal pattern were not included, as the study aimed to focus specifically on hormonal influences on sexual function.

### 2.2. Instruments

#### Sexual Function

The assessment of sexual function was conducted using the Brazilian version of the Female Sexual Function Index (FSFI). The FSFI is a 19-item instrument that assesses 6 aspects of sexual function: desire, arousal, lubrication, orgasm, satisfaction, and pain. Except for the “desire” domain, which can vary from 1.2 to 6, all the other domains have scores that vary from 0 to 6. Values <65% of that domain’s maximum score, that is, <3.9, can be considered sexual dysfunction in that domain. Although they can be assessed individually, the sum of all subscales forms the total score of this instrument, which can have scores between 1.2 and 36. Lower scores on the total score are commonly related to sexual dysfunction [17].

### 2.3. Sexual Beliefs

Sexual beliefs were assessed with the female version of the Sexual Dysfunctional Beliefs Questionnaire (SDBQ). The SDBQ is a 40-item self-reported instrument in which the individual is asked to identify their level of agreement with each statement on a 5-point Likert scale, where 1 corresponds to completely disagree and 5 to completely agree. These dimensions are as follows: 1. sexual conservatism is the dimension characterized by the idea that coitus is the central aspect of human sexuality, considering masturbation and oral and anal sex as deviant and sinful activities. A female’s sexual role is viewed as passive and receptive, with virginity being an important value for non-married women. 2. Sexual desire and pleasure as a sin is the dimension dominated by the idea that sex is a male activity, where women must control their sexual urges and pleasure since these are sinful experiences. 3. Age-related beliefs is the domain where the central theme is the decrease of sexual desire, pleasure or orgasm with age, especially after menopause. 4. Body-image beliefs is the domain characterized by the idea of body-image as a central aspect on female’s sexuality. 5. Affection primacy is the dimension where affection, love, and agreement between partners constitute the central aspect of human sexuality. 6. Motherhood primacy is the factor characterized by the idea that motherhood activities are the most important female pleasure and that procreation is the goal of any sexual experience. Although there is no cut-off point for determining the existence of sexual dysfunction in this instrument, the higher the score on the total scale (which can vary between 28 and 140 points), the greater the dysfunctional beliefs [18,19].

#### 2.3.1. Hormones

Blood samples were collected to evaluate the following hormones: luteinizing hormone (LH), estradiol, progesterone, free testosterone, sex hormone binding globulin (SHBG), prolactin, and dehydroepiandrosterone (DHEA). These analytes were selected due to their established or hypothesized roles in female sexual function during the postmenopausal period.

Blood collection took place between 7:00 and 9:00 a.m. following a 12 h overnight fast, in order to control for circadian fluctuations and ensure methodological consistency. Serum samples were used due to their greater accuracy and standardization for measuring low-concentration sex hormones in postmenopausal women. All blood collection and sample processing were conducted by a certified clinical laboratory technician with experience in research protocols. After centrifugation, serum samples were stored under temperature-controlled conditions in accordance with standard procedures for hormonal analysis. Hormone concentrations were subsequently measured using validated assay techniques. Chemiluminescence assays were used for LH, estradiol, progesterone, free testosterone, SHBG, and prolactin. DHEA was measured using radioimmunoassay (RIA), which remains the standard technique for this analyte due to its structural characteristics and low serum concentrations in postmenopausal women.

#### 2.3.2. Additional Assessments

Participants also completed the following instruments for descriptive and exploratory purposes: the Beck Depression Inventory (BDI), the Beck Anxiety Inventory (BAI), the Hospital Anxiety and Depression Scale (HAD), and the Short-Form Health Survey (SF-36). These instruments were used to assess mental health and perceived quality of life. However, as no statistically significant correlations were found between these variables and the hormonal data, their results were not included in this study.

### 2.4. Procedures

Participants who met the inclusion criteria and accepted to voluntarily participate in this study were assessed in two stages: the first stage was aimed at the application of assessment instruments; the second stage was aimed at blood collection for hormonal analysis.

The research project was approved by the Research Ethics Committee of the University Anhanguera-UNIDERP under opinion no. 505,055, also respecting ethical standards established in Resolution CNS Resolution No. 466 of 2012 of CONEP (CAAE: 17605713.2.1001.5161). All the participants signed an informed consent form.

### 2.5. Data Analysis

Descriptive data were presented as raw numbers. Categorical variables were presented using percentages and continuous variables using means and standard deviations (SDs). Data analysis was performed using the two-tailed Pearson correlation test. There were no lost values. Significance was set at *p* < 0.05. Post hoc analyses using the Bonferroni correction to adjust *p*-values were applied to the correlations between hormones and sexual beliefs. The *p*-value after the Bonferroni correction was set at *p* ≤ 0.001. Data were analyzed in the Statistical Package for the Social Sciences (SPSS) version 20.0.

## 3. Results

### 3.1. Descriptive Data

The sample consisted of 42 women who, at the time of data collection, had a mean age of (55.5 years; SD = 4.7; min. = 48; max. = 65), were educated for an average of (8.9 years; SD = 3.4; min. = 0; max. = 15), were pregnant for an average of (2.62 years; SD = 0.88; min. = 0, max. = 4) and had an average of (2.38; SD = 1.1; min. = 0; max. = 6) children. As for religion: 59.5% (*n* = 25) declared themselves as catholic, 38.1% (*n* = 16) evangelical and 2.4% (*n* = 1) as non-religious. As for marital status: 52.4% (*n* = 22) were married or in a stable relationship, 21.4% (*n* = 9) were single, 19% (*n* = 8) were divorced or separated and 7.1% (*n* = 3) were widows. As for profession: 28.6% (*n* = 12) had no profession, 23.8% (*n* = 10) were domestic workers, 11.9% (*n* = 5) worked with aesthetics, 7.1% (*n* = 3) were craftswoman or seamstresses and 28.6% (*n* = 12) had other professions. As for their occupational status: 54.8% (*n* = 23) were currently inserted in the labor market, 11.9% (*n* = 5) were retired, and 33.4% (*n* = 14) were not working in the moment of assessment. As for maternity: 97.6% (*n* = 41) had children and 2.4% (*n* = 1) had no children. As for psychological or psychiatric treatments: 69% (*n* = 29) never had any treatment, 21.4% (*n* = 9) received treatment in the past and 9.5% (*n* = 4) were undergoing treatment in the moment of the interview. The descriptive data for the general characteristics of study participants can be found in the Supplementary Table (Table S1). The sample’s descriptive data for hormonal profile, sexual function and sexual beliefs can be found in (Table 1).

### 3.2. Correlation Data

Correlations were observed between the hormones estradiol, free testosterone and prolactin and the aspect of sexual function called “desire” in the FSFI through the analysis of correlations between the examined hormones and aspects of sexual functions. A positive correlation between estradiol and desire was found (*r* = 0.337; *p* = 0.029); a positive correlation was also observed between free testosterone and desire (*r* = 0.397; *p* = 0.009); and a negative correlation was found between prolactin and desire (*r* = −0.376; *p* = 0.014).

**Figure 1:**
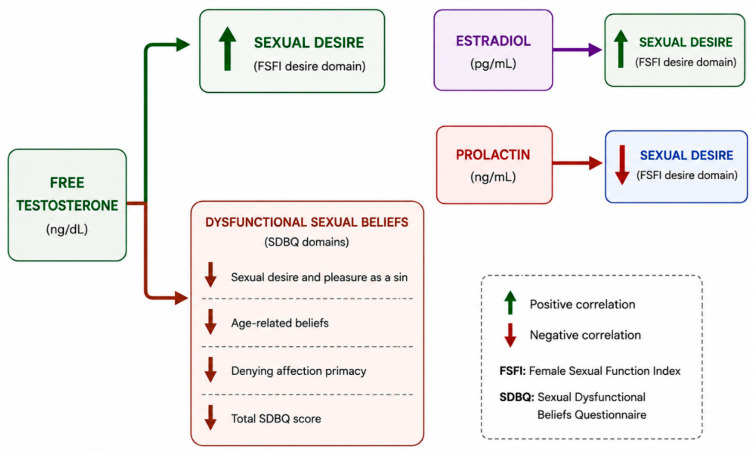
Overview of the main significant correlations between hormones, sexual desire, and dysfunctional sexual beliefs in postmenopausal women.

Regarding the correlations between sex hormones and sexual beliefs, free testosterone showed the strongest and most consistent pattern of correlations. It was negatively correlated with the belief that sexual desire and pleasure are sinful (*r* = –0.503, *p* = 0.001), age-related beliefs about declining sexual capacity (*r* = –0.400, *p* = 0.009), and beliefs denying affection as central to sexuality (*r* = –0.429, *p* = 0.005). It was also negatively correlated with the total SDBQ score (*r* = –0.432, *p* = 0.004). In addition, DHEA was negatively correlated with the domain “sexual desire and pleasure as a sin” (*r* = –0.353, *p* = 0.022), and SHBG was positively correlated with “sexual conservatism” (*r* = 0.309, *p* = 0.047) and total SDBQ score (*r* = 0.334, *p* = 0.031). All correlation results between hormones and the domains of the SDBQ can be found in (Table 2). A schematic summary of the main significant correlations observed in the present study is presented in (Figure 1).

Free testosterone was positively correlated with sexual desire (r = 0.397, *p* = 0.009) and negatively correlated with sexual desire and pleasure as a sin (r = −0.503, *p* = 0.001), age-related beliefs (r = −0.400, *p* = 0.009), denying affection primacy (r = −0.429, *p* = 0.005), and the total SDBQ score (r = −0.432, *p* = 0.004). Estradiol was positively correlated with sexual desire (r = 0.337, *p* = 0.029), and prolactin was negatively correlated with sexual desire (r = −0.376, *p* = 0.014).

Upward and downward arrows indicate the direction of statistically significant positive and negative correlations observed in the present study. Due to the cross-sectional design, these findings should not be interpreted as evidence of causal relationships.

## 4. Discussion

### 4.1. Main Findings

The study explored the relationship between hormones and sexual function in postmenopausal women not on Hormone Replacement Therapy (HRT). It found that while all domains of sexual function, as measured by the Female Sexual Function Index (FSFI), indicated dysfunction, only the “desire” domain showed significant hormonal correlations. Specifically, sexual desire was positively correlated with free testosterone and estradiol and negatively correlated with prolactin. Among all hormonal correlations observed, the association between free testosterone and sexual desire was the strongest (r = 0.397), although still within the range of weak to moderate correlations.

Additionally, the study examined sexual beliefs using the Sexual Dysfunction Beliefs Questionnaire (SDBQ). It revealed that higher levels of free testosterone were associated with fewer dysfunctional sexual beliefs, especially in the context of the belief that “sexual desire and pleasure are sinful.” This suggests that testosterone might influence not only sexual desire but also related beliefs, although further investigation is needed to clarify the direction and mechanisms of this association.

### 4.2. Interpretation

Sex hormones may influence sexual function not only through peripheral genital and vascular mechanisms but also through central neurobiological pathways involved in sexual motivation, reward processing, emotional regulation, and subjective desire. Evidence from neuroimaging studies suggests that testosterone modulates neural circuits implicated in social and affective processing, including limbic and hypothalamic regions associated with motivational and emotional responses to socially relevant stimuli [20]. Experimental studies have also demonstrated that sex steroids may promote neural plasticity through changes in dendritic spine density and synaptic connectivity in brain regions involved in reproductive and motivational behaviors [21]. In addition, growing evidence supports the concept of neurosteroidogenesis, whereby sex steroids may be locally synthesized and metabolized within the central nervous system, allowing testosterone and its metabolites to exert neuromodulatory effects on learning, memory, reproductive behavior, and other neuronal and glial functions [22]. Although much of this evidence derives from animal models and cannot be directly extrapolated to postmenopausal women, these findings provide a biologically plausible framework for understanding the associations observed in the present study between hormonal status, sexual function, and dysfunctional sexual beliefs. Nevertheless, given the cross-sectional nature of our study, these associations should not be interpreted as evidence of causality.

#### 4.2.1. Correlations Between Hormones and Sexual Function

The participants in this study presented hormone levels within the expected range for postmenopausal women not using HRT. Mean FSFI scores pointed to sexual dysfunction for all six domains of the scale.

Only the “desire” domain, one of the six aspects of sexual function assessed by the FSFI, was correlated with the hormones examined, and it is possible to observe positive correlations between desire and free testosterone as well as estradiol and a negative correlation between desire and prolactin. Considering that lower FSFI scores are indicators of sexual dysfunction, all correlations found are in agreement with the literature, since the role of testosterone on desire and estradiol as an agent in a part of this process is well known [23,24,25,26,27]. Since prolactin is negatively correlated with testosterone [28], it is expected that it is negatively correlated with desire, as observed.

The hormonal profile observed in the present sample is consistent with physiological changes associated with reproductive aging and the postmenopausal period. Menopause is characterized by ovarian follicular depletion, declining estradiol production, and compensatory increases in gonadotropins, particularly FSH. In contrast, testosterone levels tend to decline more gradually across the lifespan, allowing individual differences in androgen availability to persist after menopause. This endocrine profile may help contextualize the observed association between free testosterone and sexual desire in our sample, supporting previous evidence that androgens continue to play a clinically relevant role in female sexual function beyond the reproductive years [13,29].

From a psychological perspective, these findings reinforce the importance of considering sexuality within a broader biopsychosocial framework. Although hormonal factors may contribute to sexual well-being, previous evidence suggests that relationship characteristics and prior sexual functioning have been shown to exert a stronger influence on women’s sexual function than hormone concentrations alone. Therefore, understanding the interaction between hormonal status and psychosocial factors may help clinicians adopt more comprehensive approaches when addressing sexual complaints in postmenopausal women [13].

The strongest correlation observed between hormones and sexual function was seen between free testosterone and desire (*r* = 0.397; *p* = 0.009), which can be considered a weak correlation. In a way, this result is unexpected, since a greater influence of testosterone on desire was predicted, as reported in previous studies [23,24]. However, the less robust strength of this correlation may be explained by the fact that, in postmenopausal women, there is a significant decline in testosterone for the studied group, in addition to the fact that, despite being an important agent in modulating sexual desire, testosterone is not the only one, as sexual desire is also influenced by psychosocial factors [18]. It is also important to note that the free testosterone levels in our sample were particularly low when compared to reference ranges reported for healthy premenopausal women, a factor that may help explain the modest correlation observed between testosterone and desire. Parish et al. [29] highlight that lower androgen levels are commonly observed in women with decreased sexual interest, which is consistent with our findings.

No significant correlations were found between estradiol and other FSFI domains such as arousal, lubrication, or genital pain, which may suggest that these aspects of sexual function are more strongly influenced by non-hormonal factors in this population.

Although variables such as depression, anxiety, and quality of life were assessed, they did not show statistically significant correlations with hormonal data and were therefore not included in the main analyses. Nonetheless, the influence of unmeasured psychosocial factors in this study should be addressed in future studies.

Another important aspect to consider, based on our results, is that only the FSFI’s desire domain was correlated with some of the hormones studied, which may suggest that the relationship between hormones and sexual function is more related to sexual desire than other aspects of sexual function such as arousal, lubrication, and orgasm, with the latter being perhaps more influenced by other aspects not assessed in this study compared to desire. In this regard, our results contradict other studies that have linked testosterone to other aspects of sexuality beyond desire, such as arousal and sexual behavior [29,30,31]. This apparent dissociation between hormonal influence on desire and its lack of correlation with other behavioral aspects of sexual function may be related to the complex, multifactorial nature of female sexuality [1,2,3,4,5]. While desire is an internal psychological experience, behavioral responses such as arousal and orgasm are often mediated by contextual, relational, and emotional factors [32]—particularly relevant in postmenopausal women, who may experience physical discomfort, relational difficulties, or lack of opportunity for sexual activity.

#### 4.2.2. Correlations Between Hormones and Sexual Beliefs

The mean SDBQ scores for this sample were above those found in other studies with younger populations [16,17].

In the correlation analysis between hormones and sexual beliefs, free testosterone showed significant negative correlations with four of the six SDBQ dimensions, in addition to the total score. Higher scores in each of the dimensions of the questionnaire, as well as in the total score, are indicators of the existence of dysfunctional sexual beliefs, which in turn are related to a higher incidence of sexual dysfunctions [18,19]. The strongest correlation observed in this study was between testosterone and the dimension “Sexual desire and pleasure as a sin”, the only correlation to remain statistically significant after the Bonferroni correction (*r* = −0.503; *p* = 0.001). Thus, it is possible to consider that testosterone may influence not only on the “desire” sexual function but also sexual beliefs related to desire. While this directionality is consistent with our hypothesis and supported by the observed negative correlations, it is important to acknowledge that the relationship may be more complex and bidirectional. For this reason, we believe that other studies will need to explore this topic longitudinally.

It is important to mention that the result found in this study indicates that the higher the testosterone levels, the less dysfunctional sexual beliefs are present. This finding is noteworthy because beliefs are normally thought of as an exclusive product of culture, with little consideration being given to the hypothesis that hormones play a role in the development of beliefs. However, it is not possible to know whether this relationship occurs directly, testosterone/beliefs, or indirectly, testosterone/desire/beliefs. We believe that the hypothesis that seems more likely is an indirect relationship, and as testosterone is chronically reduced in postmenopause, it affects sexual function, and it can gradually contribute to the crystallization of previously existing dysfunctional sexual beliefs. The more intense sexual desire could act as a factor against the crystallization of a dysfunctional sexual belief. However, with reduced sexual desire and existing dysfunctional sexual beliefs related to the legitimacy of female sexual desire, the repression of the few existing desire becomes not only an easier task, but in many cases, it is culturally convenient for the moment of a woman’s life [5,33].

As we are dealing with correlation analysis, another important question should be discussed: could sexual beliefs at some level affect testosterone levels? Although few, there are studies that consider this possibility. A study that invited women to watch a movie depicting a romantic relationship between an attractive man and a woman, and in which participants were asked to watch the movie while imagining that they were the woman in the movie, found that participants’ testosterone levels increased after the movie [34]. Another study examining the effect of sexual thoughts on testosterone in women found that sexual thoughts elicit an increase in testosterone in women approximately 15 min after sexual thoughts occur [35]. Although preliminary, these findings open a promising avenue for future research and underscore the need to explore the interaction between hormonal patterns and sexual beliefs with more robust and controlled designs.

The other correlations found between hormones and sexual beliefs were weak and followed an expected pattern when considering what was specifically observed between the correlation with testosterone and sexual beliefs.

### 4.3. Strengths and Limitations

This study has several strengths. It addresses a relevant and underexplored topic by investigating the relationship between sex hormones, sexual function, and sexual beliefs in postmenopausal women—a population often neglected in research on sexuality. The inclusion of both biological and cognitive-affective dimensions allows for a more comprehensive understanding of postmenopausal sexual experience. Furthermore, the study used validated instruments and biochemical assays under controlled conditions, which increases the reliability of the data.

However, some limitations should be acknowledged. Although variables such as depression, anxiety, and quality of life were assessed, they did not show statistically significant correlations with hormonal data and were therefore excluded from the main analyses. Nonetheless, the influence of unmeasured psychosocial factors cannot be ruled out and may have contributed to the observed patterns. In addition, 9.5% of the sample was undergoing psychiatric or psychological treatment at the time of assessment. Although this subgroup represents a minority of participants and no significant associations were found between mental health indicators and hormonal data, its presence should still be acknowledged as a contextual factor potentially influencing individual variability in sexual function and beliefs.

The study also did not include variables such as smoking status, physical activity, general health conditions, history of sexual abuse, or early/premature menopause—all of which may play a role in shaping sexual behavior and beliefs. Another contextual aspect to consider is the absence of data regarding the sexual functioning of participants’ partners. While partner-related factors can influence sexual behavior and satisfaction, their impact could not be evaluated in this study and should be explored in future research.

Finally, the cross-sectional design limits the ability to establish causal relationships between the variables. Longitudinal or experimental studies are needed to explore the directionality and underlying mechanisms of the associations observed.

## 5. Conclusions

This study contributes to the understanding of how biological and cognitive-affective dimensions interact in the sexual experience of postmenopausal women. The findings suggest that testosterone may play a role not only in sexual desire but also in shaping sexual beliefs, particularly those that inhibit sexual expression. Although the associations found were modest, they highlight the need to consider hormonal and psychological aspects together, rather than in isolation, when investigating female sexual function at this life stage, which may support more comprehensive clinical assessments that integrate both physiological and psychological dimensions. For this reason and due to the limitations of this study, these results should not be considered definitive; however, they can be used as a baseline for future studies.

## Figures and Tables

**Table 1:** Descriptive sample data for hormonal profile, quality of life, mood, sexual function, and sexual beliefs.

	Variable	Minimum	Maximum	Mean	Standard Deviation
Hormones	LH (mUI/mL)	12.43	69.20	34.26	14.20
	SHBG (nmol/L)	7.90	80.60	43.56	20.18
	DHEA (ng/mL)	0.40	6.00	2.27	1.43
	Progesterone (ng/mL)	0.10	1.68	0.36	0.29
	Cortisol (µg/dL)	5.30	25.20	11.58	4.40
	Estradiol (pg/mL)	20	53	27.36	9.84
	T-free (nmol/L)	0.001	0.17	0.05	0.04
	Prolactin (ng/mL)	3.34	18.10	8.09	3.68
36-Item Short Form Health Survey (SF-36)	Functional capacity	25.00	100.00	72.38	21.36
	Limitations due to physical aspects	0.00	100.00	71.42	36.03
	Pain	20.00	100.00	55.71	17.02
	General health status	0.00	60.00	30.50	18.02
	Vitality	20.00	95.00	59.88	16.73
	Limitations due to social aspects	12.50	100.00	77.68	25.08
	Limitations due to emotional aspects	0.00	100.00	63.49	40.87
	Mental health	16.00	100.00	62.29	19.86
Mood	HAD-Anxiety	3	20	8.36	4.14
	HAD-Depression	0	16	6.12	3.29
	HAD Total score	3	32	14.48	6.36
	BAI	0	46	12.02	10.35
	BDI	4	30	13.07	6.76
Female Sexual Function Index (FSFI)	Desire	1.2	5.4	2.66	1.29
	Arousal	0.0	5.4	1.88	1.87
	Lubrification	0.0	5.7	1.87	1.94
	Orgasm	0.0	6.0	2.11	2.16
	Satisfaction	0.4	6.0	2.71	1.79
	Genital pain	0.0	6.0	2.49	2.41
	FSFI score total	1.6	33.9	13.72	9.99
Sexual Dysfunctional Beliefs Questionnaire (SDBQ)	Sexual conservatism	17	36	25.64	5.22
	Sexual desire and pleasure as a sin	6	22	13.40	3.31
	Age-related beliefs	7	20	12.43	3.13
	Body-image beliefs	3	14	8.76	2.31
	Denying affection primacy	6	18	12.02	2.05
	Motherhood primacy	8	14	9.67	1.84
	SDBQ total score	49	98	69.90	12.57

LH = luteinizing hormone; SHBG = sex hormone binding globulin; DHEA = dehydroepiandrosterone; T-free = free testosterone; HAD = Hospital Anxiety and Depression; BAI = Beck Anxiety Inventory; BDI = Beck Depression Inventory.

**Table 2:** Significant correlations between hormones and sexual beliefs.

		SHBG (nmol/L)	DHEA (ng/mL)	Progesterone (ng/mL)	Estradiol (pg/mL)	T-Free (nmol/L)
Sexual conservatism	*r*	0.309 *	−0.118	−0.185	−0.095	−0.347 *
	Sig.	0.047	0.457	0.240	0.550	0.024
Sexual desire and pleasure as a sin	*r*	0.293	−0.353 *	−0.069	−0.170	**−0.503 ^#^**
	Sig.	0.059	0.022	0.664	0.283	0.001
Age-related beliefs	*r*	0.224	−0.242	0.012	−0.234	−0.400 **
	Sig.	0.154	0.123	0.942	0.136	0.009
Body-image beliefs	*r*	0.271	−0.227	−0.100	−0.402 **	−0.191
	Sig.	0.082	0.148	0.530	0.008	0.227
Denying affection primacy	*r*	0.283	−0.262	0.331 *	−0.214	−0.429 **
	Sig.	0.070	0.094	0.032	0.173	0.005
SDBQ total score	*r*	0.334 *	−0.232	−0.104	−0.240	−0.432 **
	Sig.	0.031	0.140	0.513	0.126	0.004

SHBG = sex hormone binding globulin; DHEA = dehydroepiandrosterone; T-free = free testosterone; * Significant correlation at the level of 0.05; ** significant correlation at the level of 0.01, ^#^ significant correlation at the level of 0.001.

## Data Availability

All data generated or analyzed during this study are included in this article. Further enquiries can be directed to the corresponding author.

## Supplementary Materials

The following supporting information can be downloaded at https://www.mdpi.com/article/10.3390/jpm16070394/s1, Table S1: Descriptive data for the general characteristics of study participants.
