# Supplementary material for: Hormones, Sexual Function, and Dysfunctional Sexual Beliefs in Postmenopausal Women: A Cross-Sectional Study
Source: J Pers Med. 2026 Jul 22;16(7):394. doi: 10.3390/jpm16070394 (PMC13412903; doi:10.3390/jpm16070394)
Supplement: Supplementary file 1 [file jpm-16-00394-s001.zip › jpm-4306056-supplementary.pdf]

Table S1 - Descriptive data for the general characteristics of study participants

Category	Variable	N(%) or Mean (SD)
Demographics	Age (years)	55.5 years; SD=4.7; min.= 48; max.= 65
	Years of education	8.9 years; SD=3.4; min.=0; max.=15
	Number of pregnancies	2.62 years; SD=0.88; min.=0, max.=4
	Number of children	2.38; SD=1.1; min.=0; max.=6
Religion	Catholic	59.5% (N=25)
	Evangelical	38.1% (N=16)
	Non-religious	2.4% (N=1)
Marital status	Maried/stable union	52.4% (N=22)
	Single	21.4% (N=9)
	Divorcied/separated	19% (N=8)
	Widowed	7.1% (N=3)
Prefession	None	28.6% (N=12)
	Domestic worker	23.8% (N=10)
	Aesthetics	11.9% (N=5)
	Crafswoman/seamstress	7.1% (N=3)
	Other	28.6% (N=12)
Occupational status	Employed	54.8% (N=23)
	Retired	11.9% (N=5)
	Unemployed	33,4% (N=14)
Maternity	Has children	97.6% (N=41)
	No children	2.4% (N=1)
Psychological/Psychiatric treatment	Never	69% (N=29)
	Past	21.4% (N=9)
	Current	9.5% (N=4)
